# Web version of the protocol of the orofacial myofunctional evaluation with scores: usability and learning

**DOI:** 10.1590/2317-1782/20232022026

**Published:** 2023-04-21

**Authors:** Maria Carolina Gironde Ataide, Filipe Andrade Bernardi, Paulo Mazzoncini de Azevedo Marques, Cláudia Maria de Felício

**Affiliations:** 1 Departamento de Oftalmologia, Otorrinolaringologia e Cirurgia de Cabeça e Pescoço, Faculdade de Medicina de Ribeirão Preto - FMRP, Universidade de São Paulo - USP - Ribeirão Preto (SP), Brasil.; 2 Núcleo de Apoio a Pesquisa em Morfofisiologia Craniofacial, Universidade de São Paulo - USP - Ribeirão Preto (SP), Brasil.; 3 Programa de Pós-graduação em Bioengenharia, Universidade de São Paulo - USP - São Carlos (SP), Brasil.; 4 Departamento de Imagem Médica, Hematologia e Oncologia Clínica, Faculdade de Medicina de Ribeirão Preto - FMRP, Universidade de São Paulo - USP - Ribeirão Preto (SP), Brasil.

**Keywords:** Electronic Health Records, User-Computer Interface, Speech, Language and Hearing Sciences, Stomatognathic System, Software Validation, Registro Eletrônico em Saúde, Interface Usuário-Computador, Fonoaudiologia, Sistema Estomamatognático, Validação de Programas de Computador

## Abstract

**Purpose:**

The Orofacial Myofunctional Evaluation with Scores (OMES) protocol has been validated and used in clinical practice and research. The goals of this study were to develop, analyze and improve a version of OMES for the Web and to investigate the relationship between the usability judgments and the prior experience of the evaluators and whether using the interface promotes learning, as shown by the task completion time (TCT).

**Methods:**

Study steps: 1) inspection of the prototype by the team; 2) evaluation of usability by three experienced speech-language pathologists (SLPs); and 3) evaluation of its usability by 12 SLPs with varying levels of experience in the use of OMES. Participants answered the Heuristic evaluation (HE), the Computer System Usability Questionnaire (CSUQ), and expressed free comments. The TCT was recorded.

**Results:**

The OMES-Web reached excellent usability levels, and the participants were highly satisfied. The correlations between the participants’ experience and the HE and CSUQ scores were not significant. The TCT decreased significantly throughout the tasks.

**Conclusion:**

OMES-Web meets the usability criteria, and participants feel satisfied with the system regardless of their level of experience. The fact that it is easy to learn favors its adoption by professionals.

## RESUMO

**Objetivo:**

O Protocolo de Avaliação Miofuncional Orofacial com Escores (AMIOFE) é validado e usado na prática clínica e pesquisas. Os objetivos deste estudo foram desenvolver, analisar e aprimorar a versão do AMIOFE para a Web e investigar a relação entre os julgamentos de usabilidade e a prévia experiência dos avaliadores, e se o manuseio da interface promove o aprendizado, baseado no tempo para completar a tarefa (TCT).

**Método:**

Passos do estudo: 1) inspeção do protótipo pela equipe; 2) avaliação de usabilidade por três fonoaudiólogos experientes; e 3) avaliação de usabilidade por 12 fonoaudiólogos com variados níveis de experiência no uso do AMIOFE. Avaliação heurística (HE), Computer System Usability Questionnaire (CSUQ) e livre expressão dos participantes foram realizadas. O TCT foi registrado.

**Resultados:**

O AMIOFE-Web atingiu ótimos níveis de usabilidade e os participantes mostram-se altamente satisfeitos (Escala geral do CSUQ; Média= 1,31, DP= 0,31). A relação da experiência dos participantes e a HE e o CSUQ não foi significativa. O TCT decresceu significantemente ao longo das tarefas, mostrando que o sistema é fácil de aprender e usar.

**Conclusão:**

O AMIOFE-Web atende os critérios de usabilidade e os participantes sentiram-se satisfeitos com o sistema, independentemente de seus níveis de experiência. O fato de ser de fácil aprendizado é um fator favorável para a sua adoção por profissionais.

## INTRODUCTION

Many people are affected by impairments of the orofacial musculature, which can impact craniofacial growth and development in children, as well as orofacial function performance in all age groups^(1)^. Orofacial myofunctional disorder (OMD) is the term used to describe such impairments. Its diagnosis is based on clinical assessment, which consists of analyzing the appearance, posture, and mobility of stomatognathic system components, as well as the speech, breathing, chewing, and swallowing functions^(1-3)^. The Orofacial Myofunctional Evaluation with Scores (OMES) protocol is valid and reliable for the diagnosis of OMD in children aged 6 to 12 years^(2)^, youth, and adults^(1)^ and allows the expression of clinical data on ordinal numerical scales. Its validity is supplemented by research findings that have helped build a body of knowledge regarding orofacial myofunctional characteristics in healthy individuals and in individuals with different diseases affecting the stomatognathic system^(4-7)^. The OMES protocol is also suitable as a measure of the results of therapeutic interventions^(3,8-14)^.

The OMES protocol has been adopted in clinics, public hospitals, and higher education institutions, but it is available only in paper form. Considering that the application of information and communication technology (ICT) in healthcare can favor the retrieval of patient information, enhancing knowledge generation and the availability of this knowledge for adequate decision-making and therapeutic guidance, leading to positive consequences for health and quality of life^(15)^, a Web-based version (OMES-Web) was recently developed to fill the gap in the orofacial motricity area. There are two computerized software for assessment in the literature, namely: OMES^(16)^ and e-Myo^(17)^. However, as far as we know, an evaluation protocol web-based has not been reported.

According to ICT, before making OMES-Web available for clinical and research use, it is necessary to test its usability with an appropriate method^(18-20)^. Usability is understood as the “extent to which a system can be used by specified users to achieve specified goals with effectiveness, efficiency and satisfaction in a specified context of use”^(21)^.

Therefore, the main objective of the present study was to develop, analyze and improve the usability of OMES-Web. It was also investigated whether there was a relationship between the level of experience of the evaluator and the usability judgments, and whether using the interface promoted a learning effect.

## METHODS

### System architecture and development

The Model-View-Controller (MVC) design pattern, suitable for interfaces in Web applications^(22)^, was adopted as the architecture standard for system development. The printed OMES protocol^(2)^ was followed as a model, with some updates added and tested by the main author of the instrument^(5)^. Then the types of users and their access and use permissions were specified. The system was prepared to store evaluations by the same subject at different times or by more than one evaluator, without risk of override. For usability testing, OMES-Web was hosted on a local server managed by the research laboratory team.

### Study design and sampling overview

The study comprised three iterative steps: one involving inspection of the user interface of the prototype and the other two involving usability analysis of OMES-Web. At each step, the evaluators performed the analyses independently. The research team compiled a list of detected usability problems and suggestions from the evaluators, outlined possible solutions, and made adjustments to improve the system and its interface.

The participants were selected to represent end-users with various levels of experience in conducting evaluations using the OMES protocol. All participants were invited and gave written informed consent to participate in the study as volunteers. The Research Ethics Committee approved the study of the University Hospital of the School of Medicine of Ribeirão Preto, University of São Paulo (Process number: 013768.3.0000.5440).

To provide an experience with OMES-Web and simulate the use of a new technology in real life^(23)^, the participants were asked to transfer to the system data from previous evaluations made using the paper OMES protocol. All records belonged to a laboratory database and were coded to protect the volunteers' identity.

The usability of the interface was evaluated in steps 2 and 3 according to the “Ten principles of heuristic evaluation (HE)” proposed by Nielsen^(20)^, using the following scale: does not satisfy (score 1), partially satisfies (score 2), and satisfies (score 3). The sum of the assigned scores gave the overall score, and the higher the score, the better the usability. In step 3, the Computer System Usability Questionnaire (CSUQ)^(18)^ was also administered. It contains 19 questions and a response scale ranging from 1 (strongly agree) to 7 (strongly disagree). The general satisfaction of the user with the program was calculated by taking the mean of all CSUQ items. The three subscales represent the system usefulness, information quality, and interface quality. Lower scores indicate better usability^(18,24)^. In addition, participants were asked to express their impressions and suggestions for changes in writing. The evaluators were given explanations as to how they should proceed with HE or CSUQ. A total of three participants per group complied with the minimum number of evaluators necessary for the usability evaluation, as recommended in^(20)^.

### Step 1: Inspection of the prototype user interface

The prototype of OMES-Web was tested by members of the research group, who were instructed to transfer data from paper forms to the computer system and to provide a written description of problems related to the content, operation, and errors of the prototype. A student speech-language pathologist (SLP) previously trained to use the OMES protocol transferred data from 100 forms, and an experienced SLP also transferred 25% of these forms. The problems detected in this process were discussed with the other team members, and after a consensus, the changes were implemented.

### Step 2: First usability analysis of the OMES-Web interface

Three independent evaluators (female sex, mean age: 32.3 ± 4.7 years) analyzed the usability of OMES-Web. The selection criteria were to be an SLP with a minimum experience of five years in the clinical use of the printed OMES protocol. Each participant transferred the data from 25 printed forms via the interface, performed HE, and made suggestions in writing.

### Step 3: Second usability analysis of the OMES-Web interface

A convenience sample representing different levels of experience with the printed OMES protocol was selected and divided into groups of 3 participants each, as follows:

Group 1: Undergraduate student SLPs (n = 3, mean age ± SD = 23 ± 1.7 years) who received two training sessions;
Group 2: Undergraduate student SLPs (mean age ± SD = 23 ± 0 years) who received training during one academic year of clinical activities in orofacial motricity;
Group 3: SPLs (mean age ± SD = 34 ± 6.6 years) with a mean experience of 11.7 ± 2.5 years applying the protocol in clinical practice;
Group 4: SPLs (mean age ± SD = 32.3 ± 4.7 years) with a mean experience of 10.2 ± 4.0 years applying the protocol in clinical practice, who became familiar with the OMES-Web interface as participants in step 2.

To investigate the relationship between the usability evaluations and the participants' level of experience, these groups were given experience scores of 1, 2, 3, and 4, respectively. Because in the previous step, evaluators detected the interface problems from the first tasks, it was established that the transfer of data from five printed forms to the system would be sufficient for the analyses in step 3.

### Analysis of learning during the use of the OMES-Web interface

The time spent individually by the evaluators in transferring data from each printed form of 1 to 5 to the OMES-Web system was recorded as the task completion time (TCT). The forms' order was the same for all participants. A decrease in TCT was considered a learning effect in navigating and controlling the system during its use.

### Data analysis

HE the distribution of scores per group and the sum and percentage of the scores of all groups were calculated. The CSUQ overall satisfaction score was calculated as the mean of all items per group and the overall mean. The system usefulness, information quality, and interface quality were determined by the mean scores of specific items^(18)^. The Spearman correlation test was used to explore the relationship between the evaluators’ experience and their usability judgments. The TCTs of forms 1 to 5 were analyzed to investigate whether there was a learning effect during the use of the interface, by Friedman ANOVA and the Kendall concordance test, followed by the Wilcoxon test for tasks pairwise comparisons. The Spearman and Kendall coefficients were interpreted as weak (0.2 to 0.39), moderate (0.40 to 0.69), strong (0.70 to 0.89), or very strong (0.90 to 1.0). The statistical calculations were performed within Statistica (TIBCO Software, Inc., USA, version 13.5.013), and the level of significance was set at 5% for the tests and 1% for the post-test.

## RESULTS

### Step 1: Inspection of the prototype user interface

The prototype analysis showed problems that could affect usability. The modifications performed by the Biomedical Informatics team were related to the following problems or errors: saving the evaluations (n = 2), operation of some commands (n = 5), retrieval of information from an evaluation (n = 3), terminology (n = 9), registration information (n = 4), and the inclusion of subitems of the original protocol that were missing from the Web version (n = 5). Warnings regarding filling errors (n = 5) and options so that the evaluator could describe some findings in detail (n = 8) were also included. The values between parenthesis are corresponding to the number of changes performed. Adjustments were also implemented, such as in the interface design, organization of the database and generation of graphs according to OMES scores, and the possibility of storing photos and videos in the system.

### Step 2: First usability analysis of the OMES-Web interface

Evaluators thought that the OMES-Web completely (score 3) or partially (score 2) satisfied the heuristic usability principles. The median HE score was 27, ranging from 25 to 29, out of a total of 30 possible points. The distribution of the scores according to principle is shown in Table 1. The evaluators provided the following suggestions, which were all accepted by the team:

**Table 1:** Heuristic usability evaluation of OMES-Web. Distribution and percentage of score 3 (satisfies) assigned in steps 2 and 3 according to principle

	Step 2		Step 3					
	n = 3		G1	G2	G3	G4	Count	
			(n = 3)	(n = 3)	(n = 3)	(n = 3)	n = 12	
Principles [Nielsen (18)]	f	%	f	f	f	f	f	%
Visibility of system status	0	0	3	3	3	2	11	91.7
Match between the system and the real world	3	100	3	3	3	3	12	100
User control and freedom	3	100	3	3	3	3	12	100
Consistency and standards	1	33.3	3	2	3	2	10	83.3
Error prevention	2	66.7	3	3	3	2	11	91.7
Recognition rather than recall	3	100	3	3	2	3	11	91.7
Flexibility and efficiency of use	2	66.7	3	3	3	3	12	100
Aesthetic and minimalist design	2	66.7	3	2	2	2	9	75.0
Helps users recognize, diagnose, and recover from errors	2	66.7	3	1	2	3	9	75.0
Help and documentation	3	100	3	3	2	3	11	91.7
Total	21	70	30	26	26	26	108	90.0

Caption: n: number of participants; f: absolute frequency of response; % percentage of responses

Participants who did not assign a score of 3 gave a score of 2 (partially satisfies). No participant assigned a score of 1 (does not satisfy)

- Replace selecting the dates in the calendar with typing the dates;
- Insert fields for comments from the examiner after each of the categories (appearance/posture, mobility, and functions);
- Create an option to indicate whether the patient showed a lack of precision, tremor, or both during the assessment of mobility of the stomatognathic system components, as is usually done in the printed protocol using a mark.

### Step 3: Second usability analysis of the OMES-Web interface

Full satisfaction with OMES-Web reached 90% in step 3. Only two heuristic usability principles were judged partially satisfactory, by 25% of the evaluators, as shown in Table 1. The total score ranged from 26 to 30 points (median of 29.5). The overall CSUQ showed that participants were highly satisfied with OMES-Web, with a mean of individual scores ranging from 1.0 to 2.11 on a scale of 7 points. The system was considered highly useful, and both the information quality and interface quality were considered good (Table 2).

**Table 2:** Step 3 - CSUQ usability evaluation.

Overall	Mean	Groups (n = 12)				
		G1	G2	G3	G4	ll groups
		1.11	1.35	1.21	1.63	1.31
(1-19)	SD	0.18	0.21	0.08	0.42	0.31
System usefulness	Mean	1.13	1.33	1.17	1.54	1.29
(1-8)	SD	0.22	0.26	0.14	0.07	0.23
Information quality	Mean	1.14	1.29	1.14	1.52	1.27
(9-15)	SD	0.25	0.25	0.14	0.79	0.41
Interface quality	Mean	1.00	1.50	1.08	2.00	1.40
(16-19)	SD	0.00	0.50	0.14	0.75	0.57

Mean and standard deviation (SD) of the scores assigned by the groups. Numbers in parentheses correspond to the CSUQ items computed

Although evaluators in Group 4 assigned a score of 3 (satisfies) in the HE fewer times than those in the other groups and did not fully agree with all the CSUQ statements (score > 1), the experience of the evaluators with the OMES protocol showed a weak relationship with the HE [r = -0.28; p = 0.38; 95% confidence interval (CI): -0.73 to 0.35] and a moderate relationship with the CSUQ overall score [r = 0.50; p = 0.097; 95% CI: -0.10 to 0.83], neither significant. In this step, some evaluators provided the following suggestions, which were accepted:

- Interface design: change the interface typing font to a darker color;
- In the functional analysis of occlusion - an anthropometric measurement - exclude the predefined “0.00” notation because it overrode the measurement entered by the evaluator.

The changes made to the interface at all stages of the study resulted in the latest OMES-Web version, shown in Supplementary Material.

### Analysis of learning during the use of the OMES-Web interface

**Figure 1:**
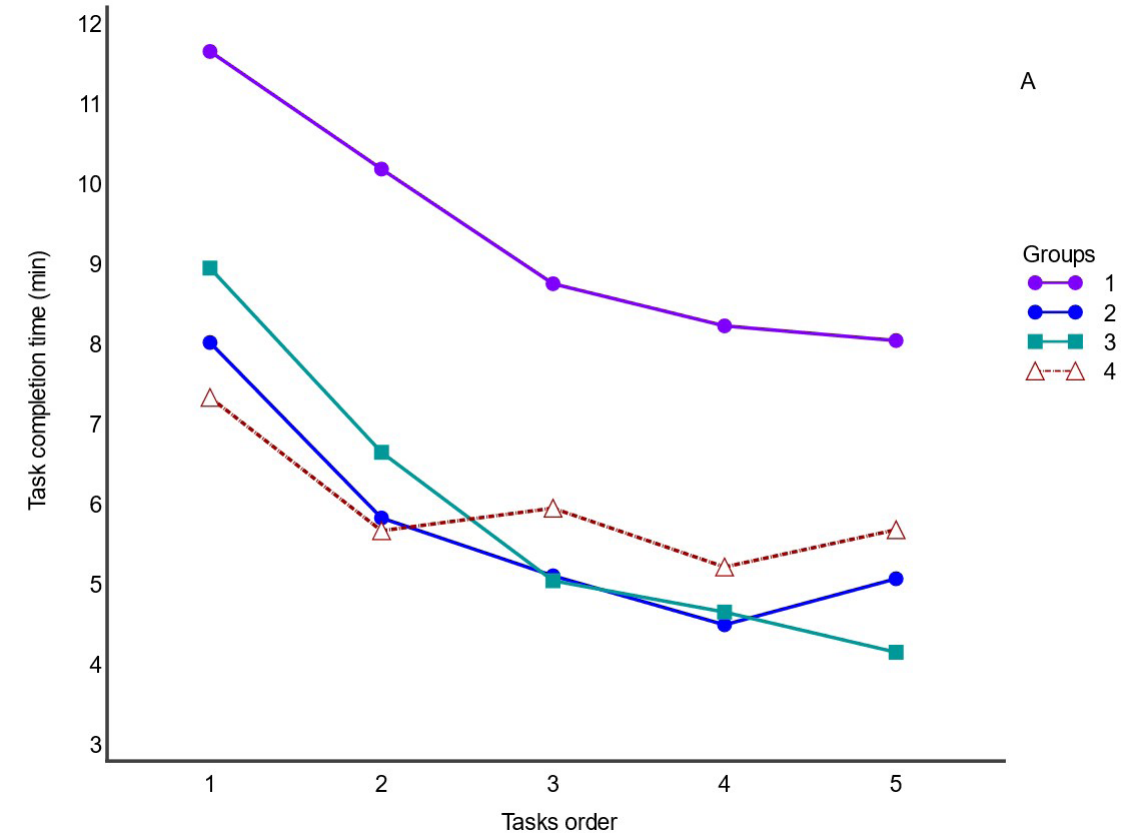
TCT per group according to the order of transfer of the protocols

Significant difference was observed in the TCTs (Friedman test, *p* < 0.0001). The TCT of the first task (median = 8.8, confidence interval CI 95% 6.6 - 11.2) was significantly longer than those of the later tasks (task 2 = 6.8, CI 95% 5.2 - 7.9; task 3 = 6.2, CI 95% 5.0 - 7.3; task 4 = 5.5 CI 95% 4.2 - 7.7; task 5 = 5.7 CI 95% 3.7 - 7.6) (Wilcoxon test, *p* < 0.01). TCT decreasing indicates a learning effect. The concordance in TCT between the different participants was moderate (Kendall coefficient = 0.58). The total time spent ranged from 22 to 53 min (mean±SD = 34.6±9.5 min). Participants with less experience (G1) spent more time on all tasks. Figure 1 illustrates the TCTs of the groups.

## DISCUSSION

In the present study, OMES-Web met the usability requirements to support its role in orofacial myofunctional evaluation. This was made possible through iterative evaluation by combining the responses to the HE^(20)^ and CSUQ^(18)^ questionnaires and the suggestions written by the evaluators, which contributed to improving it.

In the literature, integrating quantitative and qualitative methods to evaluate the usability and quality of a system is common and recommended^(19,23,25)^, as was done in the present study.

All adjustments were made with the intention of eliminating flaws and the possibility of errors that could ultimately harm patients^(26)^, in addition to making the system more efficient and the interface more pleasant in the view of its end-users, to favor its future adoption^(25,26)^.

In the first HE of the OMES-Web (step 2), the 70% full satisfaction was considered good because it was an improved prototype, but not all system resources were well defined yet. Once OMES-Web was meliorated, in step 3, it achieved 90% full satisfaction regarding heuristic usability. The main changes made were related to the visibility of the system status and consistency and standards, whose satisfaction increased from 0% and 33.33% to 91.7% and 83.3%, respectively. This indicated adequate use conditions according to the users. However, in two principles, the satisfaction percentage decreased slightly, confirming that the chance of detecting different usability problems increases when a greater number of participants perform HE^(20)^. Furthermore, considering the results of both the HE and CSUQ, not all participants were fully satisfied with the help provided by the error message or the appearance/design of the interface.

The OMES-Web error message warns if a given field was not filled out, but there is no suggestion of what to do because it was assumed that examiners would understand that they should return to it and fill it out. In addition, the warning can be disregarded if it is not possible or desirable to evaluate one or more of the items, which has the advantage of not preventing the evaluation from continuing^(16)^. However, some evaluators may have expected the system to correct them if they assigned a score that was not consistent with the patient characteristics, i.e., they did not correctly evaluate something or incorrectly marked a result. However, this was not a goal in the development of OMES-Web. Either way, if the evaluator catches their error, they can correct it before completing and saving the evaluation.

Regarding the appearance/design, the electronic protocol most likely represented a novelty, both in the way it operated and in its visual appearance, to some evaluators used to the paper version and influenced their evaluations. Group 4 was less satisfied (higher means in CSUQ) with the pleasantness of the system and with liking and feeling comfortable using it. On the one hand, professionals highly accustomed to a procedure may be more resistant to change, which becomes a barrier to the implementation of a system in health services^(27)^. On the other hand, the value of the contribution of experienced professionals in the construction and improvement of an information system seems undeniable.

Despite these observations, the participants’ experience with the OMES protocol showed no significant association with the usability of the system (HE) or overall satisfaction with it (CSUQ). This finding is relevant because a system with flexibility and efficiency of use is one that meets the needs of both experienced and inexperienced users^(20)^.

The usability of a system is also defined by its learnability, i.e., it should be easy to learn, and users can finish a job quickly using it^(20)^. OMES-Web meets this requirement, as seen by the participants’ responses to the CSUQ items related to the ease of learning and using the system. In fact, over a short time using the system, which simulated a real-life experience^(23)^, the users became familiar with and recognized its functions. Furthermore, in all groups, the TCT was higher in the first task than in the later tasks. This result is promising because being easy to use is a relevant facilitator for the adoption of a computer solution^(27)^. In addition, it suggests that, for individuals who have at least basic knowledge about the OMES protocol, a training session of approximately 60 minutes can produce enough skill to complete tasks using the system (53 min was maximal time spent by participants during all tasks). The training time needed must be considered because the successful implementation of technological solutions depends on a good strategy for training that can done in a short time^(27)^.

In the context of communication and orofacial function disorders in humans, web-based solutions have become a reality, such as programs developed for self-care after laryngectomy^(28)^, rehabilitation of persons with aphasia^(29)^, and upper airway exercises for reducing snoring^(30)^. However, as in the field of health in general^(19)^, only a portion of such applications have been tested for usability and user satisfaction^(28,29)^.

Specifically, for orofacial myofunctional evaluation, to the best of our knowledge, OMES-Web is currently the only protocol accessible by users online, which ensures ease of use and improved communication between healthcare services. Therefore, it is advantageous compared to the computerized OMES^(16)^ and e-Myo^(17)^, which were previously developed and do not offer these advantages. In addition, in the final evaluation of usability (HE), OMES-Web was superior to the computerized version, which only had 83% satisfaction and had error prevention as one of its limitations. There is no information on the validity or interface usability of e-Myo^(17)^.

OMES-Web has the potential to be used in research and clinical practice because it provides information in a clear and structured manner and facilitates access and use by the user, from the registration to the conclusion of the evaluation. Furthermore, it allows two or more evaluations of the same subject to be input in the system without risk of override and enables the creation of databases without risk of typing errors. Thus, the systematization of evaluations and the advancement of the field may be favored by OMES-Web, as it will make it possible to record, store, organize, and process data and establish comparisons that will be accessible to the multidisciplinary health team involved.

One of the limitations of this study was the number of participants, which, although sufficient for usability assessment, did not allow us to statistically analyze the effect of the participants' experience on learning. In future studies, the OMES-Web protocol should be tested in a real evaluation situation that would be influenced by several factors, including the age and clinical experience of the examiner, the dynamic between the clinician and the patient, and the location where the system is used^(23,26)^. Additionally, although its responsive platform allows it to be used in tablets or smartphones, the use of these mobile devices has certain limitations because they have smaller screens. Therefore its effectiveness must be tested before its clinical application.

## CONCLUSION

Combining the opinions expressed by evaluators with the quantitative questionnaire led to the improvement of the OMES-Web protocol for orofacial myofunctional evaluation. Above all, its usability was considered very good, and participants were highly satisfied with the system, regardless of their previous experience. Learning to navigate and control the system during its use was easy and fast. Our findings may help future research on the development of other electronic protocols and in testing OMES-Web in contexts such as teaching and learning, clinical practice, and research.

## Supplementary Material

Supplementary material accompanies this paper.

Figure S1 Initial screenshot

Figure S2 Individual information and categories of the OMES protocol for selection

Figure S3 Example of the Appearence and posture evaluation

Figure S4 Example of the lips mobility evaluation

Figure S5 Example of the occlusion analysis

This material is available as part of the online article from: https://www.scielo.br/j/codas/
