# Supplementary material for: Web version of the protocol of the orofacial myofunctional evaluation with scores: usability and learning
Source: Codas. 2023 Apr 21;35(2):e20220026. doi: 10.1590/2317-1782/20232022026 (PMC10124614; doi:10.1590/2317-1782/20232022026)
Supplement: Figure S1 [file codas-35-2-e20220026-Supl.pdf]

Supplementary Material

Usability of the Orofacial Myofunctional Evaluation with Scores protocol for the Web and analysis of the task completion time as a measure of the learning effect

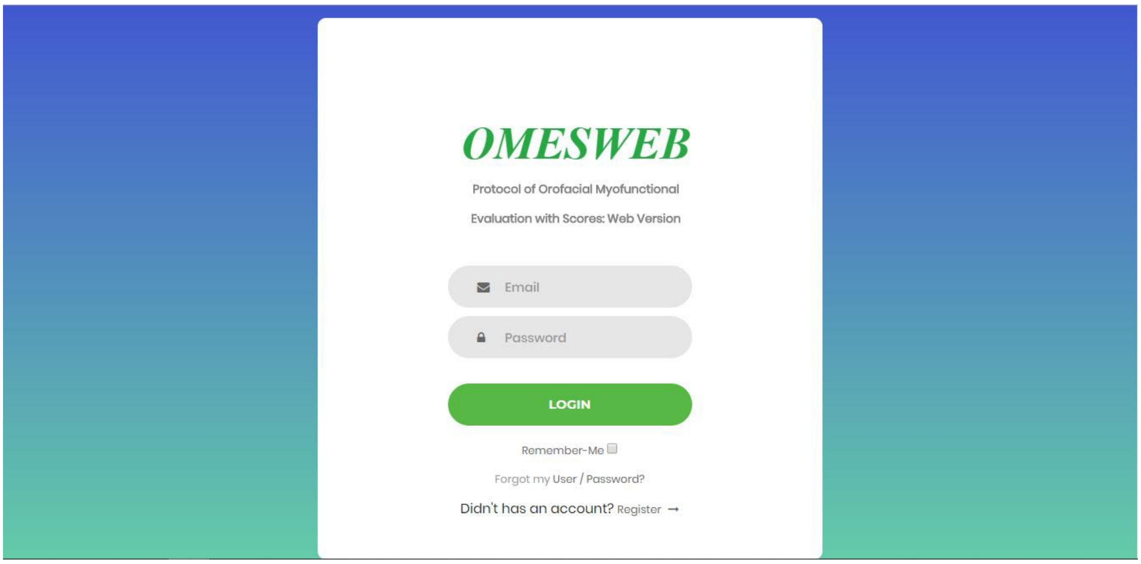

Figure S1. Initial screenshot

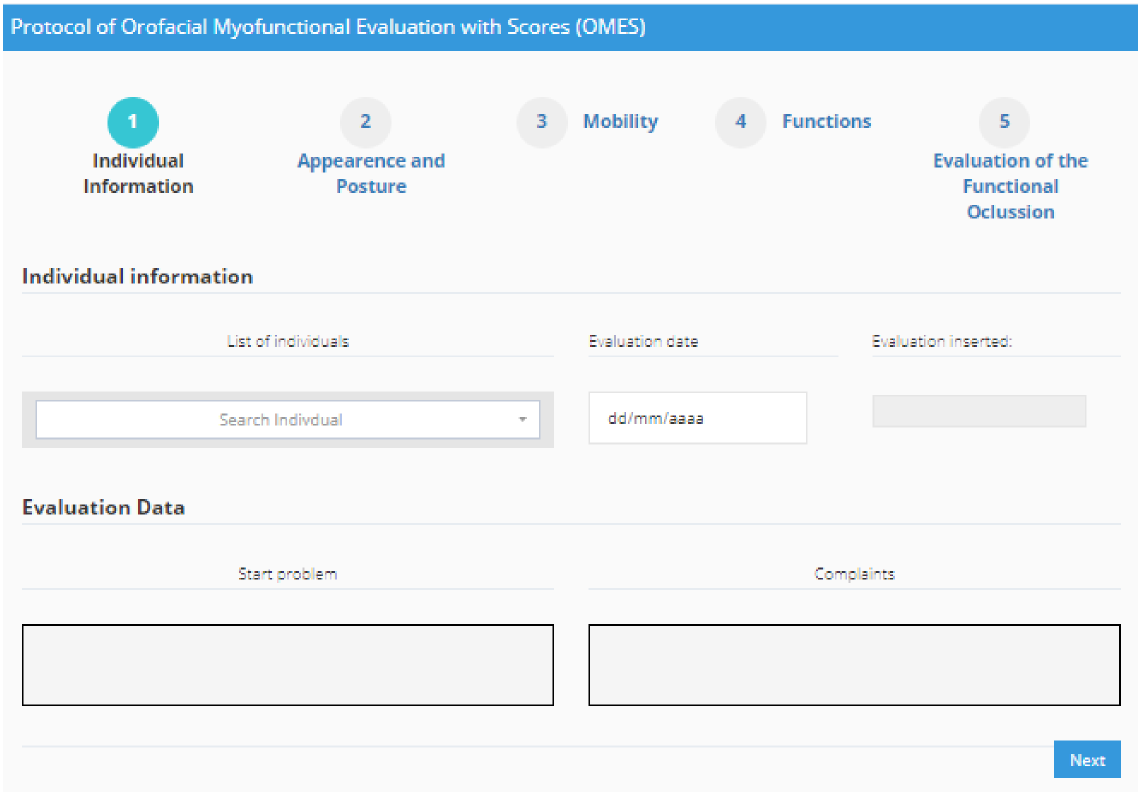

Figure S2. Individual information and categories of the OMES protocol for selection

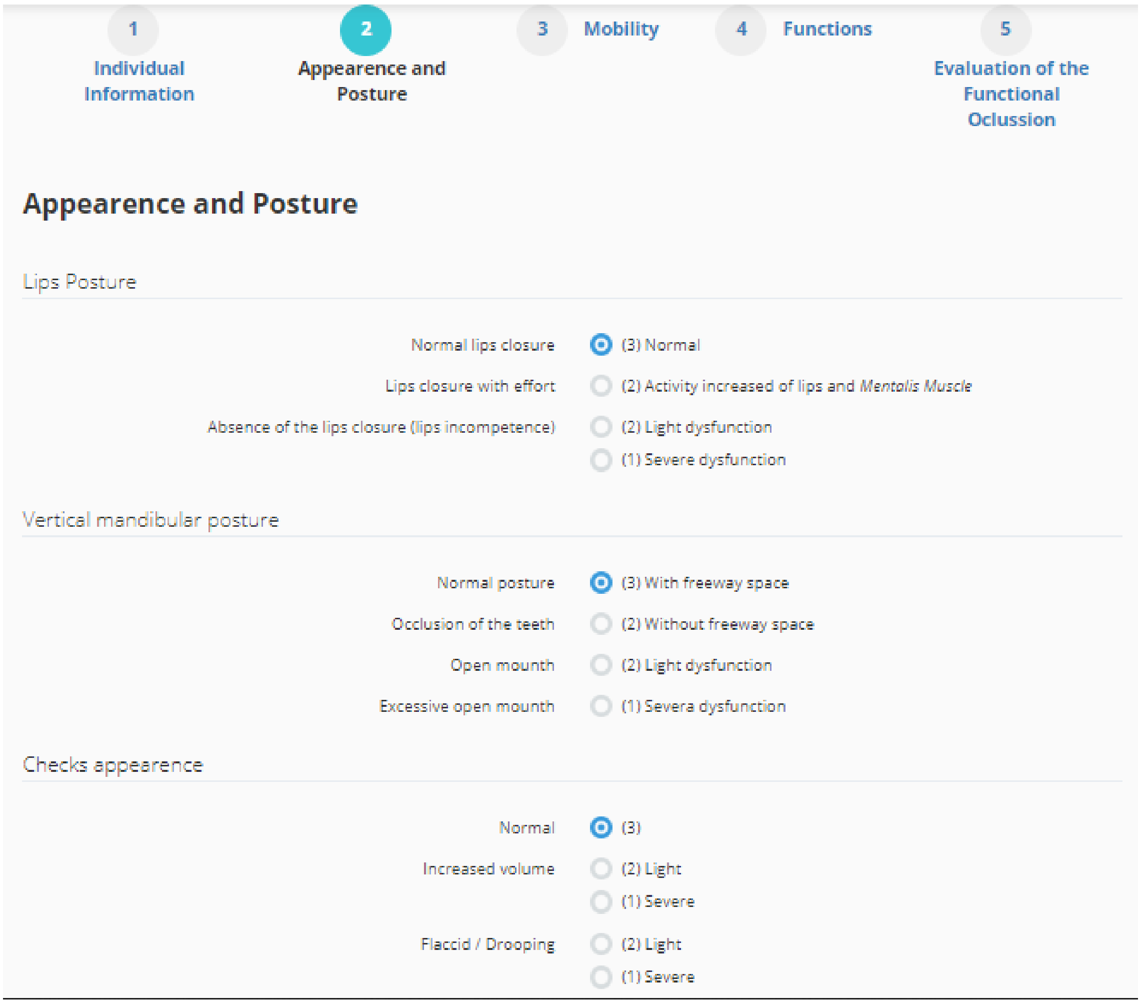

Figure S3. Example of the Appearence and posture evaluation

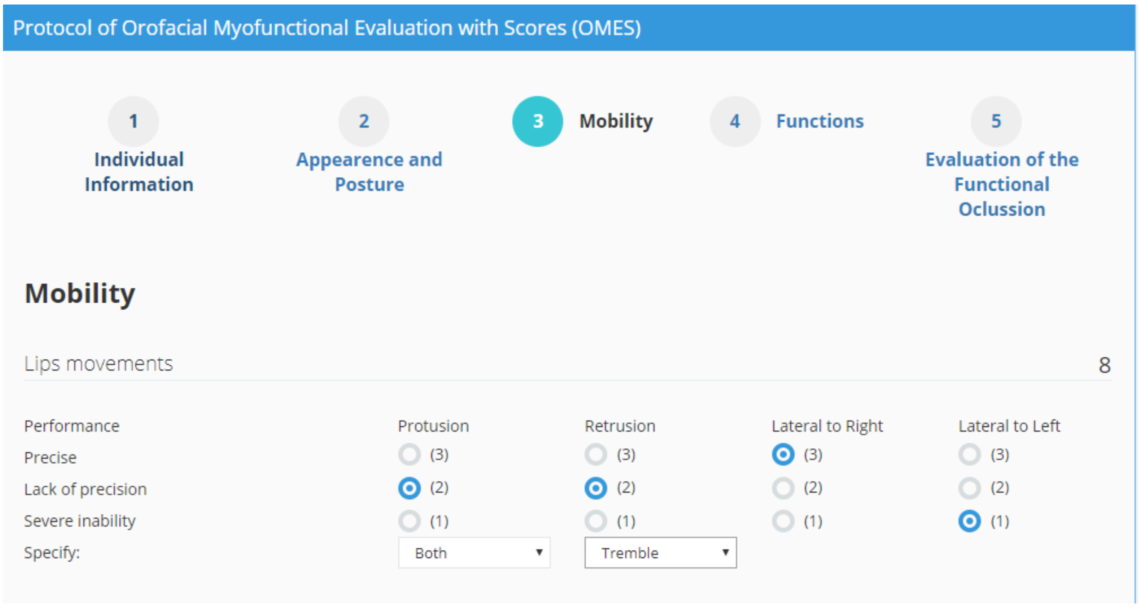

Figure S4. Example of the lips mobility evaluation

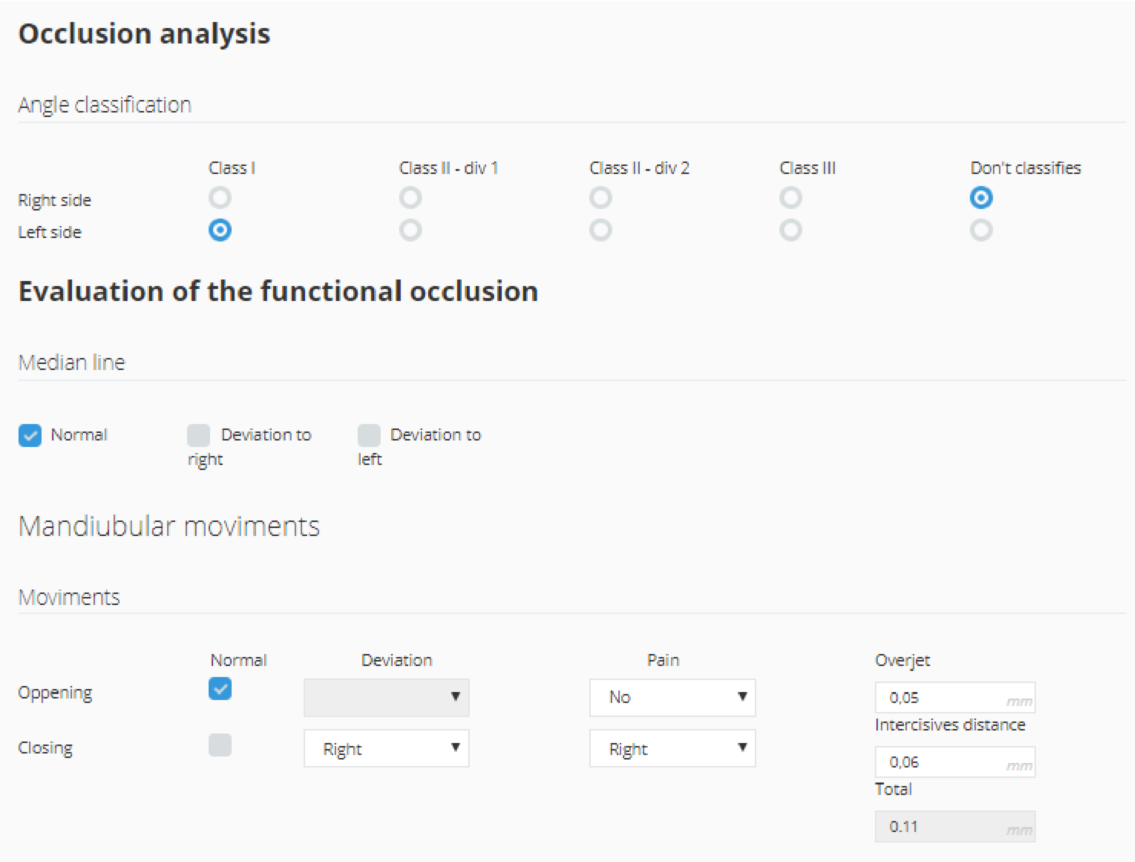

Figure S5. Example of the occlusion analysis
